# Case Report: A Novel *De Novo* Missense Mutation of the *GRIA2* Gene in a Chinese Case of Neurodevelopmental Disorder With Language Impairment

**DOI:** 10.3389/fgene.2021.794766

**Published:** 2021-11-25

**Authors:** Bingbo Zhou, Chuan Zhang, Lei Zheng, Zhiqiang Wang, Xue Chen, Xuan Feng, Qinghua Zhang, Shengju Hao, Liwan Wei, Weiyue Gu, Ling Hui

**Affiliations:** ^1^ Center for Medical Genetics, Gansu Provincial Clinical Research Center for Birth Defects and Rare Diseases, Gansu Provincial Maternity and Child Health Hospital, Lanzhou, China; ^2^ Center for Men’s Health, Gansu Provincial Maternity and Child Health Hospital, Lanzhou, China; ^3^ Chigene (Beijing) Translational Medical Research Center, Beijing, China

**Keywords:** GRIA2 gene, case report, neurodevelopmental disorder, language impairment, behavioral abnormalities

## Abstract

**Introduction:** Neurodevelopmental disorders with language impairment and behavioral abnormalities (NEDLIB) are a disease caused by heterozygous variants in the glutamate ionotropic receptor AMPA type subunit 2 (*GRIA2*) gene, which manifest as impaired mental development or developmental delay, behavioral abnormalities including autistic characteristics, and language disorders. Currently, only a few mutations in the *GRIA2* gene have been discovered.

**Methods:** A GRIA2 variation was detected in a patient by whole-exome sequencing, and the site was validated by Sanger sequencing from the family.

**Results:** We report a Chinese case of NEDLIB in a girl with language impairment and developmental delay through whole-exome sequencing (WES). Genetic analysis showed that there was a *de novo* missense mutation, c.1934T > G (p.Leu645Arg), in the *GRIA2* gene (NM_001083619.1), which has never been reported before.

**Conclusion:** Our case shows the potential diagnostic role of WES in NEDLIB, expands the *GRIA2* gene mutation spectrum, and further deepens the understanding of NEDLIB. Deepening the study of the genetic and clinical heterogeneity, treatment, and prognosis of the disease is still our future challenge and focus.

## Introduction

Neurodevelopmental disorders with language disorders and behavioral abnormalities (NEDLIB, OMIM#618917) are caused by heterozygous mutations in the glutamate ionotropic receptor AMPA type subunit 2 (*GRIA2*) gene on chromosome 4q32. The disease can lead to impaired mental development or developmental delay, poor or lack of language ability, abnormal gait, uncoordinated movement, and abnormal behaviors with autism characteristics such as stereotypes, compulsions, and repetitions. Some patients also show clinical features such as seizures and brain atrophy. The disease is onset in infancy, the clinical manifestations are highly heterogeneous, and most of them are *de novo* mutations (Salpietro et al., 2019) (Supplementary Table S1).

The glutamate receptor sensitive to α-amino-3-hydroxy-5-methyl-4-isoxazolepropionic acid (AMPA) is a ligand-activated ion channel that mediates the fast component of excitatory postsynaptic currents in neurons of the central nervous system (Krupp and Feltz, 1995; Kohda et al., 2000). The ion channel is composed of four related subunits: GLURA (*GRIA1*, OMIM*138248), GLURB (*GRIA2*, OMIM *138247), GLURC (*GRIA3*, OMIM*305915), and GLURD (*GRIA4*, OMIM*138246) (Gécz et al., 1999; Armstrong and Gouaux, 2000; Beyer et al., 2008; Allen et al., 2012). The GLURB subunit makes the channel almost impermeable to calcium ions (Ca2+) (Schoepfer et al., 1994; Utz and Verdoorn, 1997).

## Case Description

The patient and her parents went to the Medical Genetics Center of Gansu Provincial Maternity and Child Health Hospital for genetic testing. Informed consent was given according to the agreement approved by the Institutional Review Committee. The proband is a 3-year-old female whose parents were healthy and unrelated. The patient had difficulty feeding during the newborn period. Brain MRI suggested brain atrophy in the proband. She could walk independently but was slow in movement. The patient had stereotyped behavior (repeated practice of speaking) and compulsive behavior (bringing her own quilt). She did not speak continuous sentences and did not communicate with others. Her concentration was poor. She was stunted. The child had the ability to paint.

## Methods

### Whole-Exome Sequencing

DNA was obtained from peripheral blood from the patient and her parents. DNA was submitted for trio whole-exome sequencing (trioWES) to Chigene Co., Ltd. Protein-coding exome enrichment was performed using xGen Exome Research Panel v2.0 (IDT, Iowa, United States) that consists of 429,826 individually synthesized and quality-controlled probes, which targets the 39 Mb protein-coding region (19,396 genes) of the human genome and covers 51 Mb of end-to-end tiled probe space. High-throughput sequencing was performed using an MGISEQ-T7 series sequencer, and not less than 99% of the target sequence was sequenced. The sequencing process was performed by Chigene (Beijing) Translational Medical Research Center Co., Ltd.

### Bioinformatics Analysis

Raw data were processed using fastp for adapter removal and low-quality read filtering. The paired-end reads were performed using a Burrows–Wheeler Aligner (BWA) to the Ensemble GRCh37/hg19 reference genome. Base quality score recalibration together with SNP and short indel calling was conducted using GATK. According to the sequence depth and variant quality, SNPs and Indels were screened so that high-quality and reliable variants were obtained. The online system independently developed by Chigene (www.chigene.org) was used to annotate database-based minor allele frequencies (MAFs) and ACMG practice guideline–based pathogenicity of every yielded gene variant. The system also provided a series of software packages for conservative analysis and protein product structure prediction. The databases for MAF annotation include 1,000 genomes, dbSNP, ESP, ExAC, and the Chigene in-house MAF database. Provean, Sift, Polypen2_hdiv, Polypen2_hvar, Mutationtaster, M-Cap, and Revel software packages were used to predict protein product structure variation. As a prioritized pathogenicity annotation to the ACMG guideline (Richards et al., 2015), OMIM, HGMD, and ClinVar databases were used as conferences of pathogenicity of every variant. To predict functional change of variants on the splicing sites, MaxEntScan, dbscSNV, and GTAG software packages were used.

### *In silico* Analysis

Gene structure and protein domain model diagrams were performed using Illustrator for Biological Sequences v1.0 (IBS) (Liu et al., 2015). Pfam was used for protein domain prediction (Mistry et al., 2021). Protein morphology and sequence characteristics were drawn using Protter (Omasits et al., 2014). The protein 3D pictures were performed using chimeraX (Pettersen et al., 2021).

## Results

**FIGURE 1:**
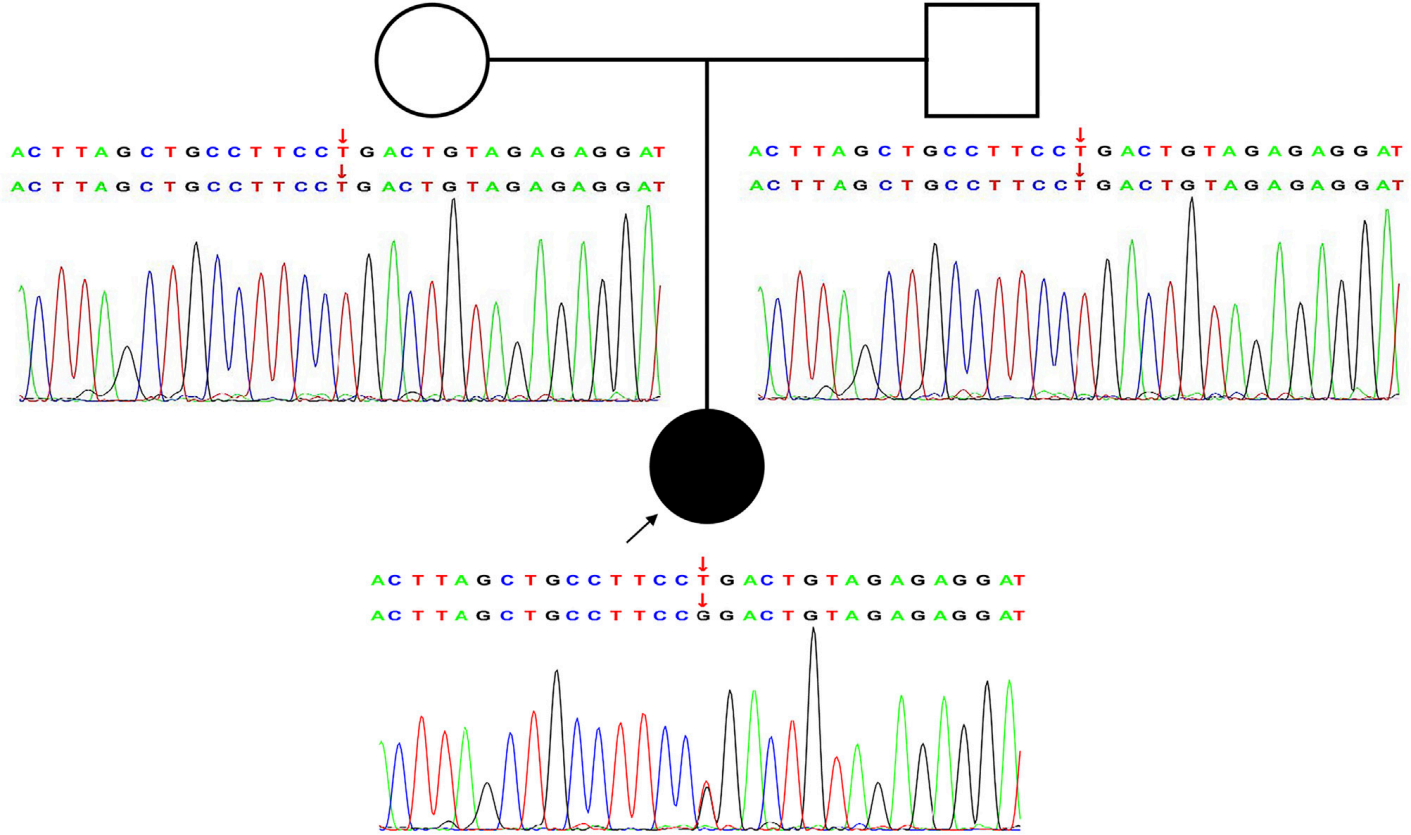
Novel heterozygous *de novo GRIA2* mutation causes NEDLIB. Pedigree and Sanger sequencing validation for the *GRIA2* NM_001083619.1: c.1934T > G (p.Leu645Arg) variant in an affected individual compared with healthy parents.

**FIGURE 2:**
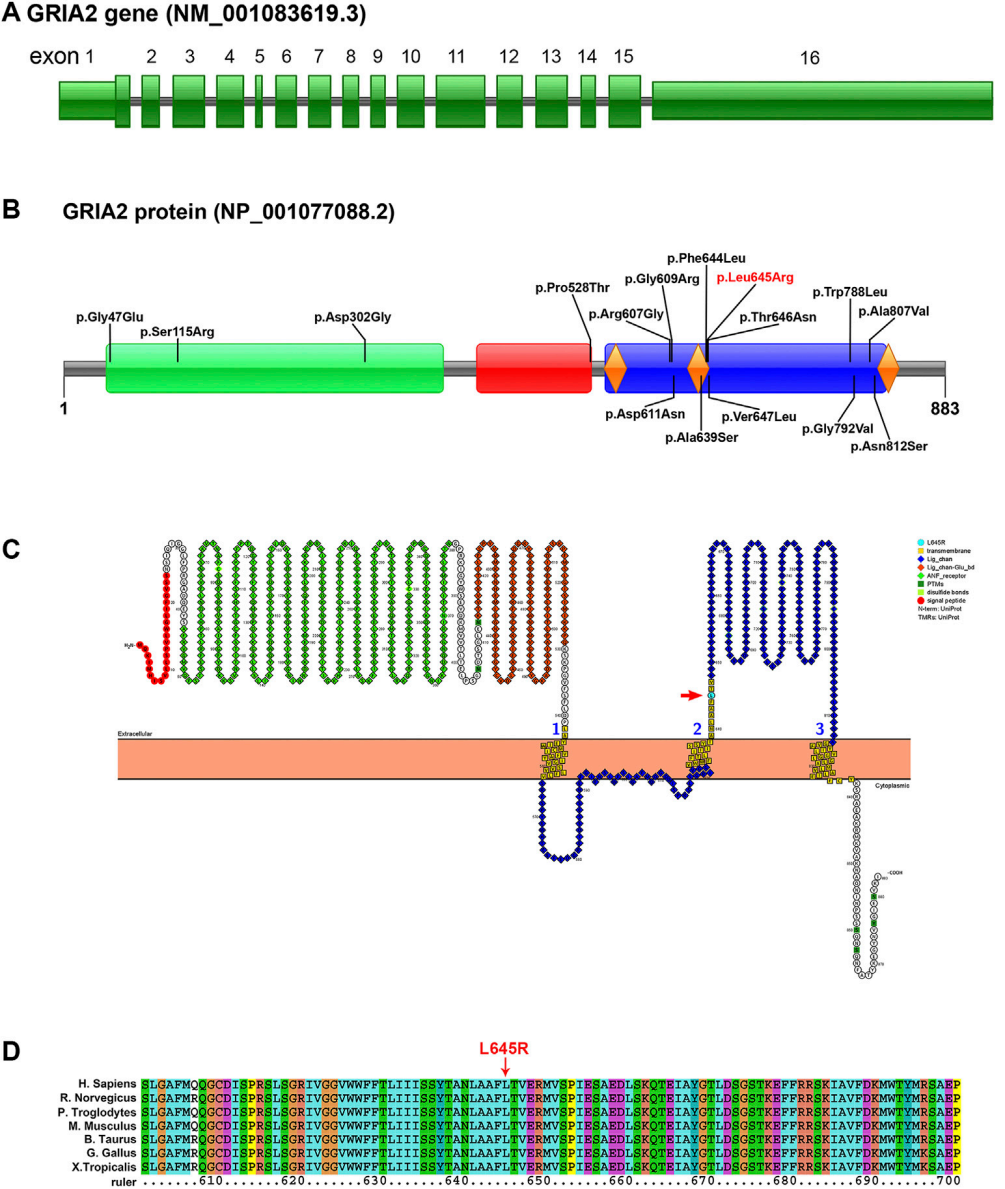
Pattern diagram of the *GRIA2* gene structure, protein domain, protein function, and transmembrane domain and protein sequence conserved analysis. **(A)** *GRIA2* gene (NM_001083619.1) contains 16 exons, and exons 2–15 are involved in gene coding. **(B)** Pattern diagram of the *GRIA2* protein domain. It shows the currently reported missense mutation sites that cause NEDLIB and the sites in this study (red font). *GRIA2* protein mainly has the following three domains: ANF_recepter (green), Lig_chan-Glu_bd (red), and Lig_chan (blue). The three transmembrane structure regions (orange diamond) are all concentrated on Lig_chan. **(C)** ANF_recepter and LIG_chan-glu_BD are located outside the cell membrane. Lig_chan had three transmembrane regions. L645R mutation site (red arrow) is in the second transmembrane region. **(D)** Multiple sequence alignment of *GRIA2* proteins from 7 species shows that the L645R mutation site is within the conserved sequence. Human (*Homo sapiens*, NP_001077088.2), Norway rat (*Rattus norvegicus*, NP_001077280.1), chimpanzee (*Pan troglodytes*, NP_001171923.2), house mouse (*Mus musculus*, NP_001077275.2), cattle (*Bos taurus*, NP_001069789.2), chicken (*Gallus gallus*, NP_001001775.2), and tropical clawed frog (*Xenopus tropicalis*, NP_001135539.1).

**FIGURE 3:**
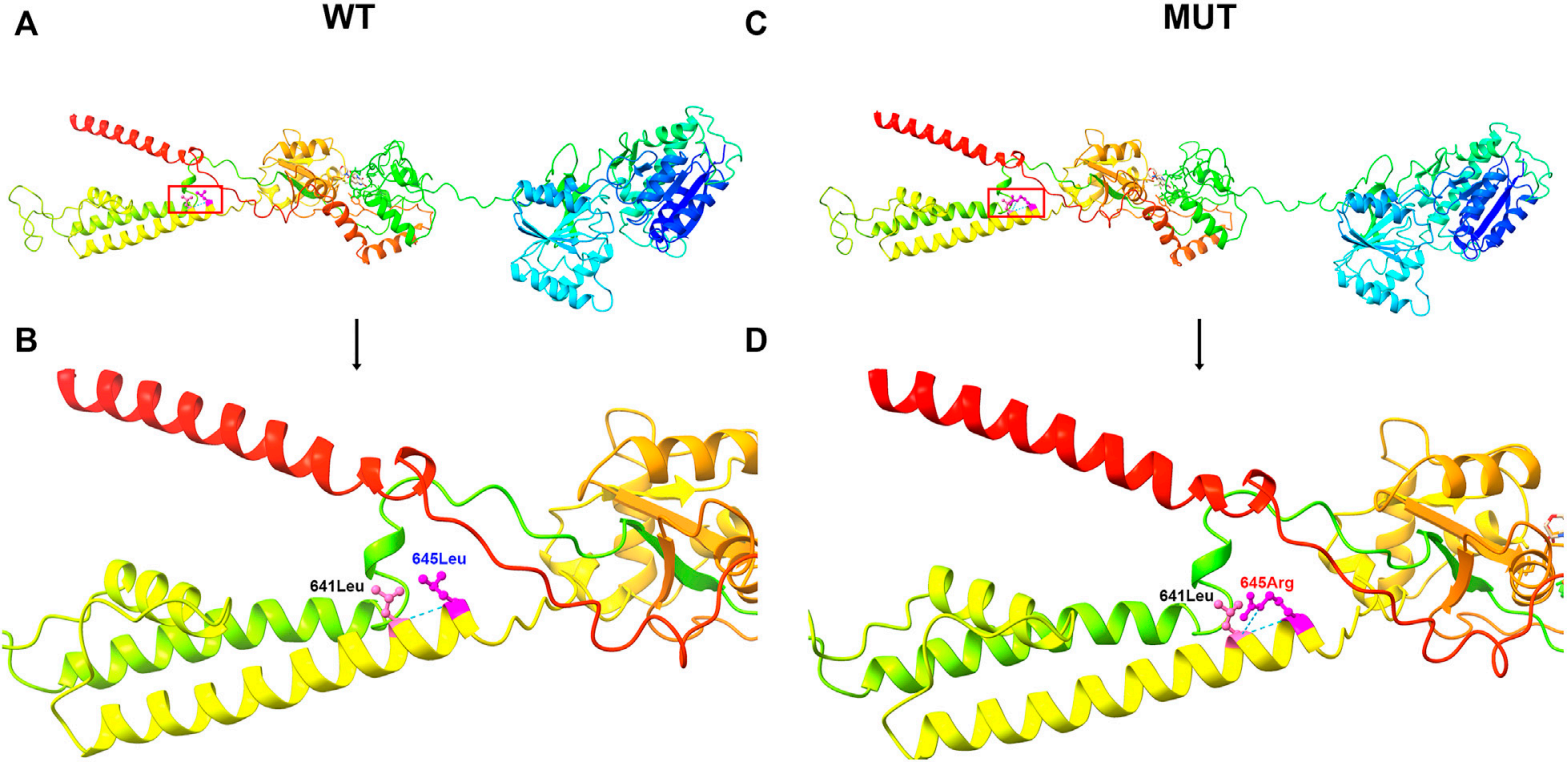
3D image of GRIA2 protein. **(A)** is the 3D overall picture of wild-type (WT) *GRIA2* protein (645Leu is in the red frame). **(B)** is the 3D partial enlarged view of wild-type *GRIA2* protein. 645Leu (WT) and 641Leu form a hydrogen bond. **(C)** is the 3D overall picture of mutant (MUT) *GRIA2* protein. **(D)** is the 3D partial enlarged view of mutant *GRIA2* protein. 645Arg (MUT) and 641Leu form two hydrogen bonds.

In this study, a heterozygous missense mutation in the *GRIA2* gene c.1934T > G (p.Leu645Arg) was detected in the proband. The mutation was *de novo*, and the parents were wild-type (Figure 1; Supplementary Table S1). According to ACMG guidelines, this variant was rated as likely to be pathogenic (PS2+PM1+PM2+PP3). The *GRIA2* gene (NM_001083619.1) contains 16 exons; part of exon 1 and exons 2–15 are involved in gene coding (Figure 2A). *GRIA2* is a transmembrane protein with three main domains: ANF_recepter (green), Lig_chan-Glu_bd (red), and Lig_chan (blue). The three transmembrane (orange diamond) areas are all on Lig_chan. So far, most of the reported missense mutations have been concentrated in the Lig_chan domain. The mutation site Leu645Arg in this study was also in this region (Figure 2B). As shown in the transmembrane protein pattern diagram, the Lig_chan (blue) domain of *GRIA2* protein (NP_001077088.2) is transmembrane three times. The Leu645Arg site (arrow) in this case is located in the second transmembrane region. Both the ANF_recepter (green) and Lig_chan-Glu_bd (red) domains are located in the extracellular region (Figure 2C). In the *GRIA2* protein sequence alignment of 7 species (human, Norway rat, chimpanzee, house mouse, cattle, chicken, and tropical clawed frog), position 645Leu is located in the conserved sequence (Figure 2D). In the 3D structure of wild-type *GRIA2* protein, 645Leu and 641Leu form a hydrogen bond (Figures 3A,B). In the 3D structure of the mutant *GRIA2* protein, Leu at position 645 became Arg (645Arg), which formed two hydrogen bonds with 641Leu (Figures 3C,D).

## Discussion and Conclusion

We report a female patient with neurodevelopmental disorders with language impairment and behavioral abnormalities (NEDLIB), presenting with delayed language development and stereotyped and compulsive behaviors. This is caused by a *de novo* missense mutation in exon 12 of the *GRIA2* gene, which has not been reported previously.

Ionic glutamate receptors (iGluRs) are ligand-gated ion channels activated by the glutamate neurotransmitter (Palmada and Centelles, 1998). iGluRs mediate most of the excitatory synaptic transmission in the central nervous system and play a key role in synaptic plasticity, which is especially important for learning and memory (Murphy-Royal et al., 2017). Gene mutations in iGluR subunits can cause developmental delay (DD), intellectual disability (ID), and autism spectrum disorders (ASDs) and other related neurodevelopmental disorders (NDDs) (Olde Loohuis et al., 2015; Moretto et al., 2018; Edfawy et al., 2019; Wilding and Huettner, 2020). According to ligand binding characteristics and sequence similarity, iGluRs are divided into 4 subtypes: AMPA receptor, alginate receptor, NMDA receptor, and delta receptor. α-Amino-3-hydroxy-5-methyl-4-isoxazole propionic acid receptors (AMPARs) are assembled from the four subunits of Glu1-4, and the most common form in the forebrain is Glu1/Glu2 heterotetramer (Herguedas et al., 2019).

The GluA2 subunit encoded by *GRIA2* plays a major role in the regulation of AMPAR Ca2+ penetration and voltage rectification. GluA2 has three main domains. The receptor family ligand binding region (ANF_receptor) includes the extracellular ligand binding domain of many receptors. This family also includes bacterial amino acid binding proteins with known structures (Kuryatov et al., 1994). The ligated ion channel L-glutamate- and glycine-binding site (Lig_chan-Glu_bd) is sometimes called the S1 domain. It is the luminal domain upstream of M1, the first transmembrane region of a transmembrane ion channel protein. This region binds l-glutamate and glycine (Ishii et al., 1993; Yamakura and Shimoji, 1999). Another time-transmembrane domain, the ligand-gated ion channel (Lig_chan) includes four transmembrane regions of ionotropic glutamate receptors and NMDA receptors (Tong et al., 1995).

Recent studies have shown that new heterozygous mutations in the *GRIA2* gene were found in 28 nonrelated neurodevelopmental abnormalities with language disorders and behavioral abnormalities, including 15 missense variants, 2 splice site variants, 1 nonsense variant, 1 in-frame deletion, and 2 frameshift mutations. These mutations occur in the entire gene. In this case, we discovered a *de novo* missense variant in the GRIA2 gene, which can lead to NEDLIB (OMIM: #138247). It is also reported that the nonsense mutation of p. Glu508Ter in the *GRIA2* gene leads to the occurrence of childhood onset schizophrenia (COS) (Alkelai et al., 2021) (Supplementary Table S1). So far, 16 missense variants of the *GRIA2* gene have been reported in 20 patients, including the p. Leu645Arg discovered this time. The phenotypes of patients with missense variants of the *GRIA2* gene are shown in Table 1. All patients have developmental delay and language impairment. A considerable number of patients have ASD and abnormal behaviors. Some patients have seizures. Conservation analysis of the amino acid sequence shows that p. Leu645Arg is in a conservative sequence. Including the p.Leu645Arg variant, 12 missense variants are concentrated in the Lig_chan domain. This domain is the transmembrane region of the *GRIA2* gene-encoding protein, which forms three transmembranes, and the p.Leu645Arg site is located in the second transmembrane region. It could be seen that the Lig_chan domain may be the hotspot mutation region of the *GRIA2* gene.

**TABLE 1:** Clinical phenotypes of patients with missense variation of the *GRIA2* gene reported to date.

Patient	Variant	Age	Gender	DD	ID	ASD	Speech impairment	Abnormal behavior	Seizures	Brain imaging	Study
1	c.140G > A; p.Gly47Glu	13 years	F	Yes	Yes	Yes	Yes	Yes	No	N/A	Salpietro et al. (2019)
2	c.345C > G; p.Ser115Arg	N/A	N/A	N/A	N/A	Yes	N/A	N/A	N/A	N/A	Satterstrom et al. (2020)
3	c.905A > G; p.Asp302Gly	10 years	M	Yes	Yes	Yes	Yes	No	No	Normal	Salpietro et al. (2019)
4	c.1582C > A; p.Pro528Thr	9 years	M	Yes	Yes	Yes	Yes	Yes	No	Normal	Salpietro et al. (2019)
5	c.1819C > G; p.Arg607Gly	11 years	F	Yes	Yes	Yes	Yes	Yes	Yes	Abnormal	Salpietro et al. (2019)
6	c.1825G > A; p.Gly609Arg	19 years	F	Yes	Yes	No	Yes	Yes	No	Yes	Salpietro et al. (2019)
7	c.1831G > A; p.Asp611Asn	19 years	M	Yes	Yes	Yes	Yes	Yes	No	Normal	Salpietro et al. (2019)
8	c.1915G > T; p.Ala639Ser	3 ms	M	Yes	N/A	N/A	N/A	N/A	Yes	Abnormal	Salpietro et al. (2019)
9	c.1915G > T; p.Ala639Ser	5 ms	F	Yes	N/A	N/A	N/A	N/A	Yes	Abnormal	Salpietro et al. (2019)
10	c.1932C > A; p.Phe644Leu	8 years	F	Yes	Yes	Yes	Yes	Yes	No	Normal	Salpietro et al. (2019)
11	c.1934T > G; p.Lys645Arg	3 years	F	Yes	N/A	No	Yes	Yes	No	Abnormal	Current study
12	c.1937C > A; p.Thr646Asn	3 years	F	Yes	N/A	No	Yes	No	Yes	Abnormal	Salpietro et al. (2019)
13	c.1939G > C; p.Val647Leu	9 years	M	Yes	Yes	N/A	Yes	No	Yes	Abnormal	Salpietro et al. (2019)
14	c.1939G > C; p.Val647Leu	5 years	M	Yes	Yes	Yes	Yes	No	Yes	Abnormal	Salpietro et al. (2019)
15	c.1939G > C; p.Val647Leu	3 years	M	Yes	Yes	N/A	Yes	No	Yes	Normal	Salpietro et al. (2019)
16	c.1939G > C; p.Val647Leu	5 years	M	Yes	Yes	N/A	Yes	No	Yes	Abnormal	Salpietro et al. (2019)
17	c.2363G > T; p.Trp788Leu	3.5 years	M	Yes	Yes	N/A	Yes	No	Yes	Abnormal	Salpietro et al. (2019)
18	c.2375G > T; p.Gly792Val	31 years	F	Yes	Yes	Yes	Yes	Yes	No	N/A	Salpietro et al. (2019)
19	c.2420C > T; p.Ala807Val	3.6 years	F	Yes	Yes	Yes	Yes	Yes	Yes	Normal	Salpietro et al. (2019)
20	c.2435A > G; p.Asn812Ser	3 years	M	Yes	Yes	Yes	Yes	Yes	No	Normal	Salpietro et al. (2019)

DD, developmental delay; ID, intellectual disability; ASD, autism spectrum disorder; ys, years; ms, months.

Taken together, our research has expanded the *GRIA2* gene mutation spectrum, indicating that the detection and diagnosis of NEDLIB through WES is feasible and effective.

## Data Availability

The original contributions presented in the study are included in the article/Supplementary Material; further inquiries can be directed to the corresponding author.
