# Supplementary material for: Case Report: A Novel De Novo Missense Mutation of the GRIA2 Gene in a Chinese Case of Neurodevelopmental Disorder With Language Impairment
Source: Front Genet. 2021 Nov 25;12:794766. doi: 10.3389/fgene.2021.794766 (PMC8655903; doi:10.3389/fgene.2021.794766)
Supplement: Supplementary file 1 [file Table1.DOCX]

Supplementary Table 1. GRIA2 intragenic variants associated with NEDLIB identified in previously report and current study.

| **Patient** | **Genomic coordinates (GRCh37/hg19)** | **Transcript** | **Variant** | **SIFT** | **PolyPhen** | **Mutation Taster** | **disease** | **author** | **year** |
| --- | --- | --- | --- | --- | --- | --- | --- | --- | --- |
| 1 | 4:158282233-158282233 | NM001083619.1 | c.2363G>T; p.Trp788Leu | D(0) | D(0.999) | D(1) | Intellectual disability, seizures and speech impairment | Salpietro | 2019 |
| 2 | 4:158257636-158257645 | NM_000826.3 | c.1582_1590del; p.Pro528_Lys530del | - | - | - | Intellectual disability, seizures and speech impairment | Salpietro | 2019 |
| 3 | 4:158257886-158257886 | NM_000826.3 | c.1831G>A; p.Asp611Asn | D(0.01) | D(0.999) | D(1) | Intellectual disability, seizures and speech impairment | Salpietro | 2019 |
| 4 | 4:158257880-158257880 | NM_000826.3 | c.1825G>A; p.Gly609Arg | D(0) | D(1) | D(1) | Intellectual disability, seizures and speech impairment | Salpietro | 2019 |
| 5 | 4:158253993-158253993 | NM_000826.3 | c.905A>G; p.Asp302Gly | D(0) | D(0.78) | D(1) | Intellectual disability, seizures and speech impairment | Salpietro | 2019 |
| 6 | 4:158282773-158282773 | NM_000826.3 | c.2375G>T; p.Gly792Val | D(0) | D(1) | D(1) | Intellectual disability, seizures and speech impairment | Salpietro | 2019 |
| 7 | 4:158257874-158257874 | NM_000826.3 | c.1819C>G; p.Arg607Gly | D(0) | B(0.054) | D(0.99) | Intellectual disability, seizures and speech impairment | Salpietro | 2019 |
| 8 | 4:158142284-158142284 | NM_000826.3 | c.88+2T>C; p.? | - | - | - | Intellectual disability, seizures and speech impairment | Salpietro | 2019 |
| 9 | 4:158257637-158257637 | NM_000826.3 | c.1582C>A; p.Pro528Thr | D(0) | D(0.999) | D(1) | Intellectual disability, seizures and speech impairment | Salpietro | 2019 |
| 10 | 4:158257839-158257840 | NM_000826.3 | c.1785del; p.Phe595LeufsX37 | - | - | - | Intellectual disability, seizures and speech impairment | Salpietro | 2019 |
| 11 | 4:158257900-158257900 | NM_000826.3 | c.1844+1G>A; p.? | - | - | - | Intellectual disability, seizures and speech impairment | Salpietro | 2019 |
| 12 | 4:158242725-158242726 | NM_000826.3 | c.857del; p.Pro286LeufsX14 | - | - | - | Intellectual disability, seizures and speech impairment | Salpietro | 2019 |
| 13 | 4:158262508-158262508 | NM_000826.3 | c.1937C>A; p.Thr646Asn | D(0) | D(0.998) | D(1) | Intellectual disability, seizures and speech impairment | Salpietro | 2019 |
| 14 | 4:158262503-158262503 | NM_000826.3 | c.1932C>A; p.Phe644Leu | D(0) | D(0.99) | D(1) | Intellectual disability, seizures and speech impairment | Salpietro | 2019 |
| 15 | 4:158142870-158142870 | NM_000826.3 | c.140G>A; p.Gly47Glu | B(0.14) | D(0.942) | D(1) | Intellectual disability, seizures and speech impairment | Salpietro | 2019 |
| 16 | 4:158262510-158262510 | NM_000826.3 | c.1939G>C; p.Val647Leu | D(0) | D(0.994) | D(1) | Intellectual disability, seizures and speech impairment | Salpietro | 2019 |
| 17 | 4:158262486-158262486 | NM_000826.3 | c.1915G>T; p.Ala639Ser | D(0.01) | D(0.997) | D(1) | Intellectual disability, seizures and speech impairment | Salpietro | 2019 |
| 18 | 4:158262510-158262510 | NM_000826.3 | c.1939G>C; p.Val647Leu | D(0) | D(0.994) | D(1) | Intellectual disability, seizures and speech impairment | Salpietro | 2019 |
| 19 | 4:158254055-158254055 | NM_000826.3 | c.967C>T; p.Arg323ter | - | - | - | Intellectual disability, seizures and speech impairment | Salpietro | 2019 |
| 20 | 4:158262486-158262486 | NM_000826.3 | c.1915G>T; p.Ala639Ser | D(0.01) | D(0.997) | D(1) | Intellectual disability, seizures and speech impairment | Salpietro | 2019 |
| 21 | 4:158262510-158262510 | NM_000826.3 | c.1939G>C; p.Val647Leu | D(0) | D(0.994) | D(1) | Intellectual disability, seizures and speech impairment | Salpietro | 2019 |
| 22 | 4:158283979-158283979 | NM_000826.3 | c.2435A>G; p.N812S | D(0) | D(0.994) | D(1) | Intellectual disability, seizures and speech impairment | Salpietro | 2019 |
| 23 | 4:158282726-158282726 | NM_000826.3 | 2328G>T; p.Glu776Asp | (D)0.02 | (D)0.994 | D(1) | Intellectual disability, seizures and speech impairment | Salpietro | 2019 |
| 24 | 4:158262510-158262510 | NM_000826.3 | c.1939G>C; p.Val647Leu | D(0) | D(0.994) | D(1) | Intellectual disability, seizures and speech impairment | Salpietro | 2019 |
| 25 | 4:157362812-157362812 | NM_000826.3 | c.2420C>T; p.A807V | D(0.01) | D(0.886) | D(1) | Intellectual disability, seizures and speech impairment | Salpietro | 2019 |
| 26 | 4:158257577-158257577 | NM_000826.3 | c.1522 G > T; p.Glu508Ter | - | - | - | childhood onset schizophrenia | Alkelai | 2021 |
| 27 | 4:158262505-158262505 | NM_000826.3 | c.1934T>G; p.Leu645Arg | D(0) | D(0.999) | D(1) | developmental disorders, speech impairment, abnormal Behavior | this study | 2021 |
