# Supplementary material for: Case Report: A Novel De Novo Missense Mutation of the GRIA2 Gene in a Chinese Case of Neurodevelopmental Disorder With Language Impairment
Source: Front Genet. 2021 Nov 25;12:794766. doi: 10.3389/fgene.2021.794766 (PMC8655903; doi:10.3389/fgene.2021.794766)
Supplement: Supplementary file 2 [file DataSheet1.PDF]

CARE
case report guidelines

CARE Checklist of information to include when writing a case report

(cc) BY-NC-ND

Topic	Item	Checklist item description	Reported on Line
Title	1	The diagnosis or intervention of primary focus followed by the words "case report"	1 - 2
Key Words	2	2 to 5 key words that identify diagnoses or interventions in this case report, including "case report"	14 - 15
Abstract (no references)	3a	Introduction: What is unique about this case and what does it add to the scientific literature?	34 - 49
	3b	Main symptoms and/or important clinical findings	51 - 59
	3c	The main diagnoses, therapeutic interventions, and outcomes	93 - 110
	3d	Conclusion—What is the main "take-away" lesson(s) from this case?	154 - 155
Introduction	4	One or two paragraphs summarizing why this case is unique (**may include references**)	138 - 153
Patient Information	5a	De-identified patient specific information.	51 - 59
	5b	Primary concerns and symptoms of the patient.	51 - 59
	5c	Medical, family, and psycho-social history including relevant genetic information	51 - 59
	5d	Relevant past interventions with outcomes	51 - 59
Clinical Findings	6	Describe significant physical examination (PE) and important clinical findings.	51 - 59
Timeline	7	Historical and current information from this episode of care organized as a timeline	51 - 59
Diagnostic Assessment	8a	Diagnostic testing (such as PE, laboratory testing, imaging, surveys).	62 - 70
	8b	Diagnostic challenges (such as access to testing, financial, or cultural)	62 - 70
	8c	Diagnosis (including other diagnoses considered)	62 - 70
	8d	Prognosis (such as staging in oncology) where applicable	not applicable
Therapeutic Intervention	9a	Types of therapeutic intervention (such as pharmacologic, surgical, preventive, self-care)	not applicable
	9b	Administration of therapeutic intervention (such as dosage, strength, duration)	not applicable
	9c	Changes in therapeutic intervention (with rationale)	not applicable
Follow-up and Outcomes	10a	Clinician and patient-assessed outcomes (if available)	not applicable
	10b	Important follow-up diagnostic and other test results	not applicable
	10c	Intervention adherence and tolerability (How was this assessed?)	not applicable
	10d	Adverse and unanticipated events	not applicable
Discussion	11a	A scientific discussion of the strengths AND limitations associated with this case report	112 - 153
	11b	Discussion of the relevant medical literature **with references**.	112 - 153
	11c	The scientific rationale for any conclusions (including assessment of possible causes)	112 - 153
	11d	The primary "take-away" lessons of this case report (without references) in a one paragraph conclusion	154 - 155
Patient Perspective	12	The patient should share their perspective in one to two paragraphs on the treatment(s) they received	not applicable
Informed Consent	13	Did the patient give informed consent? Please provide if requested	Yes ☑ No ☐
